# Spatiotemporal immune gradients in gout: immune response–driven activation of the NLRP3–IL-1β axis and its transition to trained immunity

**DOI:** 10.3389/fimmu.2026.1776479

**Published:** 2026-02-27

**Authors:** Kang Wang, Jiabin Li, Jing Li, Fan Zeng, Siren Li, Pei Chen, Hui Xiong

**Affiliations:** 1 Orthopedics Department, The First Hospital of Hunan University of Chinese Medicine, Changsha, China; 2 Department of Orthopedics, Huaihua City Hospital of Traditional Chinese Medicine, Huaihua, China; 3 Graduate School, Hunan University of Chinese Medicine, Changsha, China; 4 Department of Orthopedics, The Affiliated Hospital of Jiangxi University of Traditional Chinese Medicine, Nanchang, China

**Keywords:** gout, immune response, inflammatory response, precision therapy, spatiotemporal immune gradient, trained immunity

## Abstract

Gout is a crystal-associated autoinflammatory disease triggered by monosodium urate (MSU) crystals, clinically characterized by recurrent transitions between acute inflammatory flares dominated by innate immunity and a state of “trained immunity” during the remission phase. However, previous studies have mostly focused on single time points or local lesions. Such approaches fail to systematically explain the recurrent nature of acute gout flares and the mechanisms underlying multi−system involvement. By integrating evidence from single-cell and spatial transcriptomics as well as mechanistic investigations, this review systematically summarizes the immunopathological features of gout within a spatiotemporal immune framework. At the temporal level, acute gout flares are driven by innate immune activation of the NOD-like receptor pyrin domain-containing protein 3 (NLRP3)–interleukin-1β (IL-1β) inflammatory cascade. The inflammation then undergoes self-limited resolution mediated by regulatory T cells (Tregs), M2-polarized macrophages, aggregated neutrophil extracellular traps (aggNETs), and pro-resolving lipid mediators. persistent low-grade activation of monocytes/macrophages can still be observed, sustaining a state of “trained immunity.” At the spatial level, integrated evidence indicates an immune gradient across the joint, bone, and circulation, ranging from focal hyper-inflammation to systemic low-grade activation. Based on these findings, we propose a time-window stratified intervention strategy centered on the NLRP3–IL-1β axis, and identify inflammatory markers in the joints, subchondral bone, and peripheral blood as the basis for spatially targeted stratification. These insights provide novel perspectives for shifting gout management from the control of individual flares to recurrence risk management and personalized therapy.

## Graphical Abstract

**Figure:**
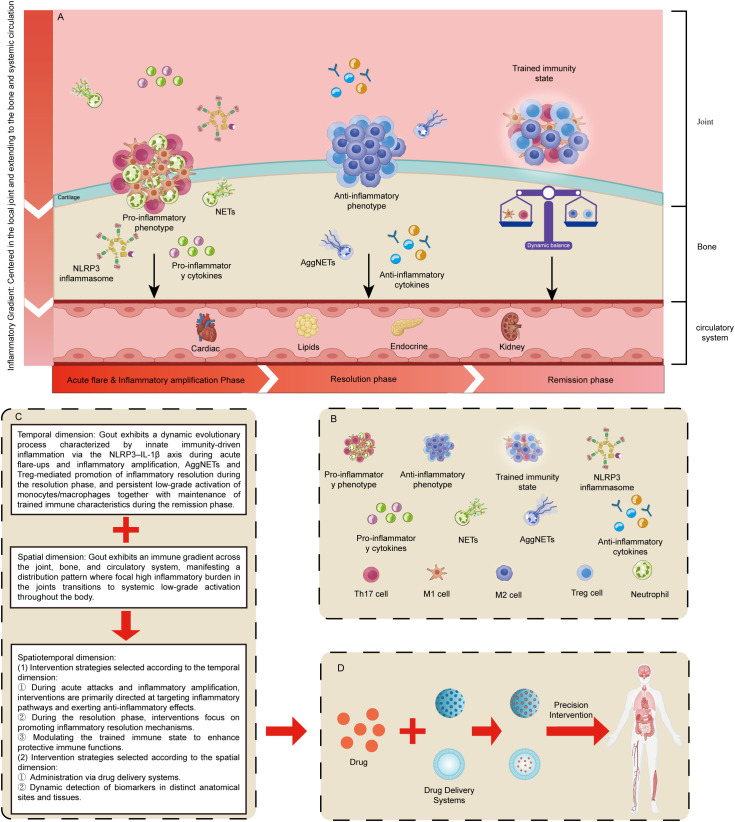
**(A)** Spatiotemporal model of immune responses in gout: The horizontal axis depicts the temporal dimension of gout progression, spanning the acute flare phase, inflammation amplification phase, resolution phase, and remission phase. The vertical axis represents a spatial gradient extending from the local joint to the skeletal system and the systemic circulation. During the flare and amplification phases, the dominant mechanisms are pro-inflammatory cascades mediated by M1-polarized macrophages, Th17 cells, and NETs, accompanied by robust activation of the NLRP3 inflammasome. Upon entry into the resolution phase, immune lineages undergo functional reprogramming, with M2-polarized macrophages, Tregs, and aggNETs driving active inflammation resolution. The remission phase reveals features of “trained immunity, “ primarily involving monocyte and T-cell subsets, characterized by a dynamic immune homeostasis maintained under conditions of low-grade activation. **(B)** Identification of immune cell subsets and molecular components: This panel provides detailed symbol definitions and terminological annotations for the key immune cell subsets, signaling molecules, and microenvironmental components depicted in the figure. **(C)** Spatiotemporal framework of gout and stratified intervention strategies: This panel integrates temporal disease evolution with spatial inflammatory gradients in gout. Based on this framework, “time-oriented” strategies targeting distinct inflammatory stages and “space-oriented” precision interventions targeting specific anatomical compartments are proposed, aiming to shift clinical management from global anti-inflammatory approaches to precision intervention. **(D)** Drug delivery systems and drug conjugation: A schematic illustration of drug delivery systems conjugated with therapeutic agents, enabling targeted delivery to lesion sites to achieve precise intervention and optimized therapeutic efficacy.

## 1 Introduction

Gout is a prevalent inflammatory arthritis triggered by the deposition of monosodium urate (MSU) crystals within joints and periarticular tissues. Its global prevalence varies markedly, ranging from 0.1% to 10%, and the disease exhibits a consistent trend toward increasing incidence and progressively younger age at onset (1). Clinically, gout classically presents with acute onset of erythema, swelling, warmth, and severe pain in affected joints. The underlying pathological mechanism is directly attributable to hyperuricemia, which promotes the local formation and deposition of MSU crystals, thereby eliciting a vigorous inflammatory cascade (2). If left uncontrolled long-term, these crystals continuously erode cartilage, bone, and tendons, leading to progressive joint destruction and functional loss, which represents the primary cause of disability in gout (3). The clinical course of gout is characterized by alternating cycles of acute flares and clinical remission. This cyclic pattern is rooted in the complex immunopathophysiological mechanisms of the disease. The progression from hyperuricemia to MSU crystal formation and subsequent acute inflammatory flares involves a delicate and intricate cascade of immune and inflammatory responses. As endogenous danger signals, MSU crystals are directly recognized by innate immune cells, including macrophages and neutrophils, and activate the intracellular NOD-like receptor pyrin domain-containing protein 3 (NLRP3) inflammasome. This process leads to the release of the pro-inflammatory cytokines interleukin-1β (IL-1β) and IL-18 (4).

In recent years, breakthroughs in cutting-edge technologies such as single-cell sequencing and spatial transcriptomics have greatly advanced our understanding of the dynamic behavior of the immune system in disease (5, 6). Emerging evidence indicates that, across diverse pathological conditions, including inflammation, cancer, and autoimmunity, immune cells do not constitute a homogeneous or static population. Instead, they exhibit marked heterogeneity in phenotype, function, and spatial distribution across distinct disease stages and anatomical sites (7, 8). This spatiotemporal dynamics of the immune response is critical in determining disease initiation, progression, resolution, and recurrence. It also offers novel perspectives and theoretical foundations for the development of mechanism−stratified precision interventions.

**Figure 1:**
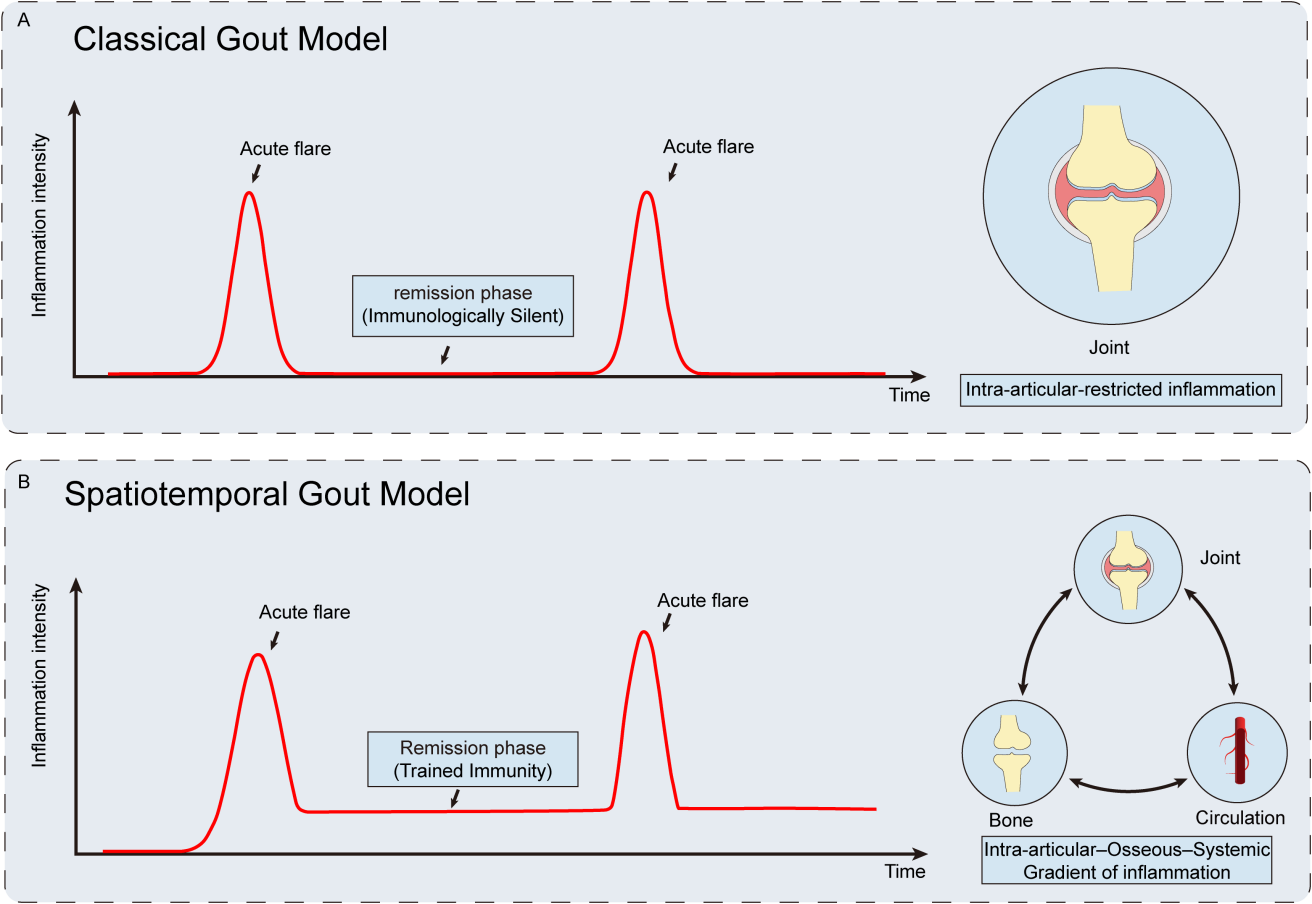
Comparison between the classical view and the spatiotemporal model of gout. **(A)** The classical view conceptualizes gout as a series of isolated acute flares, with the remission phase regarded as an immunologically quiescent state and inflammation confined to the joint. **(B)** In contrast, the proposed “spatiotemporal” model indicates that inflammation persists during remission in a low-grade activated state characterized by features of trained immunity. In this framework, gout is viewed as a systemic inflammatory gradient involving dynamic interactions among the joint, the skeletal system, and the systemic circulation, rather than a purely localized joint disease.

Gout, as a typical inflammatory disease with alternating acute and chronic phases, exhibits a profound spatiotemporal pattern in its immune response. This review primarily focuses on the most characteristic clinical stages of gout: the acute attack phase and the clinical remission phase. The “spatiotemporal immune gradient” described herein refers to the systematic and continuous changes in the immune response across temporal (e.g., flare phase, inflammation amplification phase, resolution phase, remission phase) and spatial (from the joint as the core, extending to bone and circulation) dimensions during the progression of gout. This gradient integrates multiple interconnected layers, including the distribution of immune cell subsets, cellular activation states, dynamic changes in key cytokines and chemokines, as well as the transcriptional and epigenetic features of immune cells, and emphasizes the interplay between temporal dynamics and spatial heterogeneity. Based on this conceptual framework, this review aims to systematically elucidate the immune response characteristics during the acute and remission phases of gout, with a particular focus on the temporal patterns of immune response over time and the spatial immune gradient that spans the joint, bone, and circulation. Finally, we will explore how this spatiotemporal immune framework can guide the shift toward a precision intervention model in the diagnosis and treatment of gout, emphasizing time-layered and space-directed approaches (**Figure 1**).

## 2 Immune response during the acute phase of gout

Acute gout flares are driven by an intense sterile inflammatory response triggered by the deposition of MSU crystals in the joint and surrounding soft tissues (9). When MSU crystals are recognized by the immune system as endogenous danger signals, they initiate complex innate and adaptive immune responses, which directly underlie the clinical manifestations of the acute phase of gout (10, 11). The physicochemical properties of urate crystals, including their size, morphology, and surface charge, markedly influence their pro-inflammatory potential. In addition, crystal deposition activates the complement system, leading to the generation of complement component 3a (C3a) and C5a, induces necrotic forms of cell death, and stimulates the production of acute-phase proteins, such as C-reactive protein, thereby collectively amplifying the local inflammatory milieu (12).

### 2.1 Changes in immune cell phenotypes and functions

MSU crystals can be phagocytosed by neutrophils and monocytes/macrophages, leading to the activation of the intracellular NLRP3 inflammasome and the release of large amounts of pro-inflammatory mediators (13). Among these cells, macrophages occupy a central position. Evidence indicates that tissue-resident macrophages may initiate and drive the early inflammatory response, whereas recruited monocyte-derived macrophages are more actively involved in the subsequent resolution of inflammation (14). MSU crystals induce metabolic–inflammatory reprogramming in macrophages, involving pathways related to lipid and amino acid metabolism, glycolysis, oxidative stress, and apoptosis (12). Activated macrophages produce cyclooxygenase-2 (COX-2), nuclear factor-κB (NF-κB), and key cytokines, including interleukin-1β (IL-1β), tumor necrosis factor-α (TNF-α), IL-6, and IL-18, thereby further amplifying the inflammatory response (12). In addition to phagocytosis, neutrophils contribute to inflammation by undergoing necrosis or forming neutrophil extracellular traps (NETs), releasing MSU crystals and NETs components, which sustain and exacerbate local inflammatory responses (15). In the context of adaptive immunity, T-lymphocyte subsets also play important roles. T helper 17 (Th17) cells promote neutrophil recruitment through the secretion of interleukin-17 (IL-17) in MSU-induced inflammatory environments and are linked to receptor activator of nuclear factor-κB ligand (RANKL)-mediated osteoclastogenesis. In contrast, regulatory T cells (Tregs) suppress excessive immune responses by producing anti-inflammatory factors, including IL-4, IL-10, and transforming growth factor-β1 (TGF-β1). Th1 and Th2 cells contribute to immune regulation through the secretion of cytokines such as interferon-γ (IFN-γ), IL-2, and IL-18, and IL-4 and IL-10, respectively. Notably, certain T-cell subsets can also express RANKL, thereby directly promoting osteoclast formation and participating in bone erosion (16). In addition, changes in immune cell growth, differentiation, and functional states profoundly influence the inflammatory course during the acute phase (17).

### 2.2 Activation of the NLRP3 inflammasome

Activation of the NLRP3 inflammasome represents a central molecular event in MSU crystal–triggered inflammation during the acute phase of gout (4). Acting as danger signals, MSU crystals promote the assembly of the NLRP3 inflammasome, leading to the activation of caspase-1, which subsequently cleaves pro–IL-1β and pro–IL-18 into their biologically active forms, IL-1β and IL-18, thereby initiating and amplifying the inflammatory cascade (18). The signaling regulation of this process involves multiple mechanisms. The signaling regulation of this process involves multiple mechanisms. The generation of reactive oxygen species (ROS) not only directly contributes to the activation of the NLRP3 inflammasome but also influences the activation of related signaling pathways, including nuclear factor erythroid 2–related factor 2 (Nrf2) and the myeloid differentiation primary response 88–nuclear factor-κB (MyD88–NF-κB) axis (17). Notably, uric acid itself, beyond its crystalline form, can potentiate MSU-induced pro-inflammatory responses. Clinical observations indicate that serum IL-1β levels are significantly elevated in patients with hyperuricemia and gout and show a positive correlation with serum urate concentrations, further supporting a critical role for uric acid in inflammasome activation and the pathogenesis of gout (19).

### 2.3 Inflammatory cascade and cytokines

**Figure 2:**
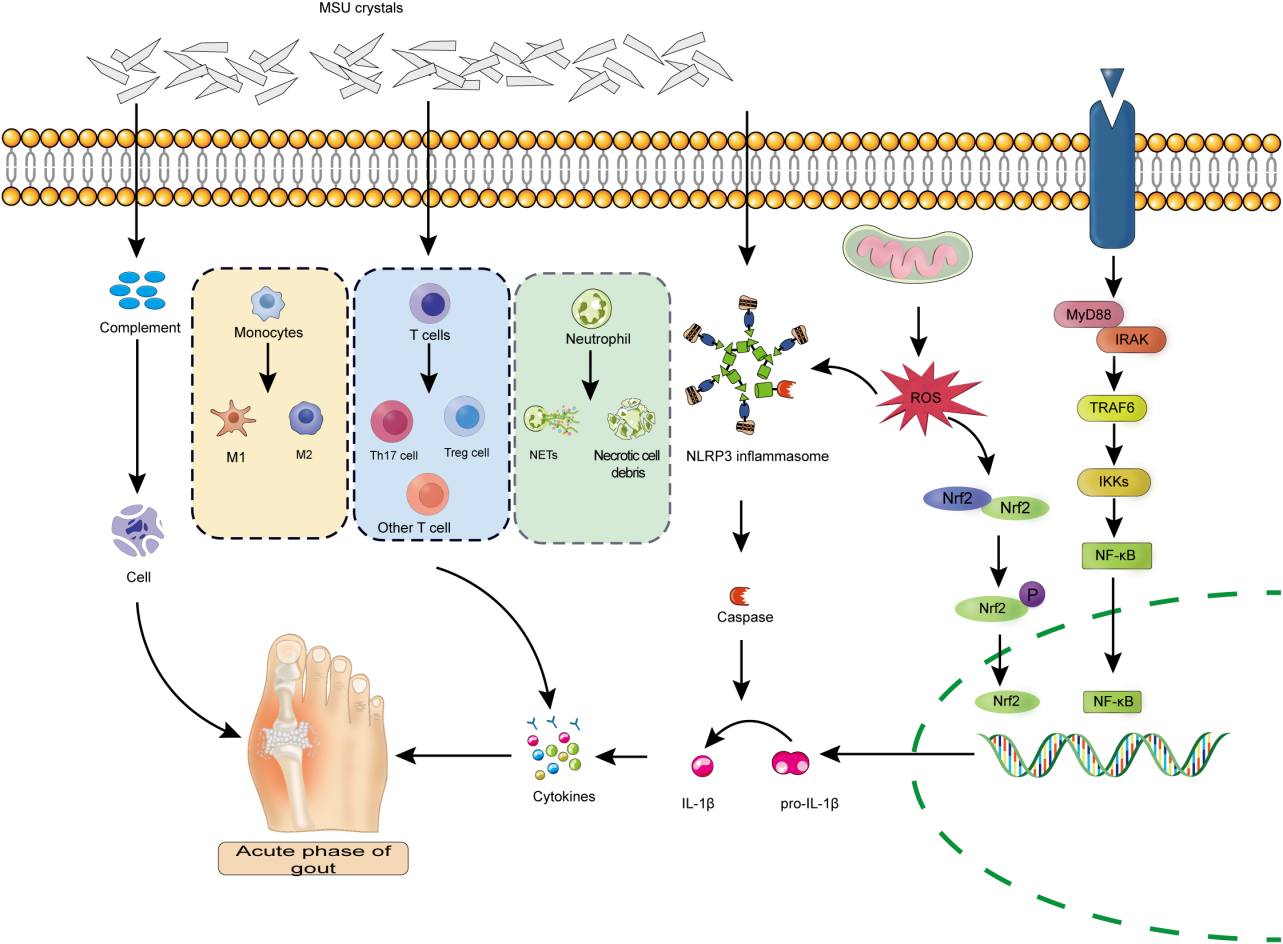
Cellular and molecular mechanisms underlying MSU crystal–induced inflammation during the acute flare of gouty arthritis: The figure illustrates how MSU crystals activate immune cells through multiple pathways to trigger an inflammatory storm. Neutrophils, monocytes, and T-cell subsets (such as Th17 and Tregs) recognize MSU crystals and initiate large-scale immune responses, including the formation of NETs and the release of pro-inflammatory cytokines. MSU crystals also activate the complement system and exacerbate local tissue damage through necrotic cell death pathways. In addition, MSU crystals promote the assembly of the NLRP3 inflammasome, leading to activation of caspase-1, which cleaves the precursor pro-inflammatory cytokines pro–IL-1β and pro–IL-18 into their bioactive forms, IL-1β and IL-18, for extracellular release. Moreover, ROS modulate activation of the MyD88/NF-κB signaling pathway to enhance pro-inflammatory gene transcription and influence Nrf2-mediated antioxidant responses. Collectively, these pathological processes form a self-amplifying inflammatory loop that ultimately gives rise to the clinical manifestations of acute gout.

The inflammatory response triggered by MSU is a complex cascade process that involves the coordinated actions of multiple cytokines and inflammatory mediators (20). Among these, cytokines such as IL-1β, IL-6, IL-8, IL-17, IL-18, and TNF-α play central roles in gout-associated inflammation (21). For example, serum levels of IL-1β and IL-9 are significantly elevated in patients with gout and show a positive correlation with serum urate concentrations (19). In addition, chemokines, reactive oxygen species, components of the complement system (such as C-reactive protein), and myeloperoxidase are also broadly involved in, and further amplify, local inflammatory responses (22). Although acute gout flares are typically self-limiting and resolve spontaneously within days to weeks, the resolution of inflammation is not a passive process but instead depends on active regulatory mechanisms. This process involves the production of multiple anti-inflammatory mediators, such as IL-37, which effectively restrains MSU crystal–induced innate immune responses and promotes the resolution of inflammation (23) (**Figure 2**).

## 3 Immune responses during the chronic remission phase of gout

Although the intense inflammatory responses characteristic of the acute phase of gout have been extensively investigated (4), the immune state during the clinical remission phase is equally complex and critically important. This stage does not represent a simple state of inflammatory “quiescence”. Instead, it reflects a dynamic equilibrium shaped by persistent MSU crystal deposition, low-grade immune activation, the establishment of trained immunity, and imbalances in active resolution mechanisms. A deeper understanding of the immunological features of the remission phase is essential for explaining disease recurrence, developing long-term management strategies, and ultimately achieving clinical cure.

### 3.1 Persistent MSU crystal deposition and sustained immune activation

The clinical remission phase does not represent the pathological endpoint of gout. Evidence indicates that MSU crystal deposition in the joint and surrounding tissues persists during remission and continues to provide chronic stimulation to the immune system, driving a state of subclinical inflammation that underlies disease recurrence and chronic progression (24). Deposited MSU crystals can be recognized and phagocytosed by immune cells such as tissue-resident macrophages. This process sustains intracellular NLRP3 inflammasome activation and maintains a local pro-inflammatory milieu at a low level (25). This chronic stimulation, together with its precursor soluble uric acid, may induce persistent functional and epigenetic reprogramming of innate immune cells. Seminal studies have demonstrated that soluble uric acid can “train” primary human monocytes, enabling them to mount enhanced inflammatory responses, including increased IL-1β production, upon subsequent stimulation, through specific epigenetic regulatory mechanisms (26). Based on such evidence, a theoretical framework has been proposed suggesting that a “trained immunity–like” state may exist in gout (27). This state is thought to lower the activation threshold of the immune system, thereby providing a molecular-level hypothesis to explain the propensity for recurrent acute gout flares (28). This renders the immune system more sensitive to subsequent crystal stimuli and lowers the response threshold, providing a potential molecular-level explanation for the recurrent nature of acute gout flares (25).

### 3.2 Dynamic remodeling of immune cell phenotypes and functions

During the remission phase, the persistent deposition of MSU crystals drives phenotypic and functional remodeling of immune cells, forming the cellular basis of subclinical inflammation and the risk of disease recurrence. monocytes/macrophages do not revert to a fully resting state during remission. Instead, the NLRP3 inflammasome pathway remains at a low level of activation, leading to sustained production of pro-inflammatory mediators. Single-cell transcriptomic studies suggest that this persistent low-grade activation is accompanied by remodeling of cellular phenotypes (6). Within the theoretical framework of trained immunity, this state is thought to render immune cells more responsive to subsequent stimuli, thereby potentially increasing the risk of disease recurrence (27, 29). At the same time, although neutrophils do not infiltrate in large numbers as observed during the acute phase, they can still contribute through low-level NETosis, releasing residual crystals and pro-inflammatory mediators that help maintain the local inflammatory microenvironment within the joint (15, 30). The adaptive immune system also undergoes key alterations. Single-cell RNA sequencing (scRNA-seq) analyses have demonstrated the presence of MSU crystal–associated, disease-specific transcriptomic changes in peripheral blood mononuclear cells (PBMCs) from patients during the remission phase (24). Among these changes, the balance between pro-inflammatory Th17 cells and anti-inflammatory Tregs is critical. Th17 cells exacerbate inflammation and may drive bone erosion through the secretion of cytokines such as IL-17, whereas Tregs attempt to restrain immune responses by producing IL-10 and TGF-β1. Disruption of this balance represents a key step in the transition from remission to disease relapse (16). More recent studies further reveal that the suppressed state of specific lymphocyte subsets, such as Vδ2 T cells, is closely associated with stable clinical remission and may serve as a potential biomarker for predicting disease recurrence (31). In addition, the contribution of B cell–mediated humoral immunity has increasingly attracted attention, as it may participate in chronic inflammatory processes through the production of autoantibodies (32). This crystal-driven chronic stimulation, leading to widespread activation and dysregulation of both the innate and adaptive immune systems, collectively defines the unstable immunological state characteristic of the remission phase of gout.

### 3.3 Trained immunity and “threshold” regulation of gout recurrence

The clinical remission phase of gout essentially represents a state of subclinical immune activation shaped by trained immunity. In this state, immune cells maintain a “pre-activated” phenotype even after the initial stimuli have subsided, enabling a faster and more robust response to subsequent danger signals, such as MSU crystals (33). Similar mechanisms have been described in inflammatory arthritis, where pronounced immune cell dynamics can still be observed during remission and are closely linked to the risk of disease relapse (34). In the context of gout, the persistent presence of MSU crystals, fluctuations in local joint pH and temperature, or concomitant infectious events may all serve as secondary stimuli. When the intensity of these stimuli exceeds the individualized immune relapse threshold shaped by trained immunity, an uncontrolled inflammatory cascade is rapidly initiated, resulting in an acute flare. Accordingly, the remission phase of gout can be viewed as a “threshold period” characterized by the dynamic accumulation of relapse risk (35).

### 3.4 Mechanisms of inflammation resolution during the remission phase of gout

The resolution of inflammation during the remission phase of gout is not a state of passive quiescence but rather an active and programmed biological process, centered on restoring immune microenvironmental homeostasis and initiating tissue repair (36). Following an acute flare, the host activates a series of intrinsic pro-resolving mechanisms to precisely terminate inflammatory responses. These mechanisms include the downregulation of key pro-inflammatory signaling pathways, such as NF-κB; the biosynthesis of bioactive specialized pro-resolving lipid mediators (SPMs), including lipoxins, protectins, resolvins, and maresins; the generation of non-lipid pro-resolving mediators; the efficient clearance of apoptotic cells and debris through efferocytosis; and the induction of immunoregulatory Tregs (37). For example, the local production of anti-inflammatory cytokines such as IL-10 and Annexin A1 (ANXA1) effectively suppresses further neutrophil chemotaxis and infiltration, thereby actively promoting the resolution of inflammation (38). However, during the remission phase of gout, this finely tuned resolution program may not be fully or durably restored. Insufficient activation or premature termination of these pathways can result in incomplete resolution, allowing local or systemic tissues to remain in a state of chronic, low-grade inflammation. Such an “unresolved” microenvironment may not only directly contribute to ongoing tissue damage but is also thought to increase susceptibility to acute gout flares, thereby driving the recurrent cycle of flare and remission.

### 3.5 Trained immunity: a potential mechanism requiring further validation

**Figure 3:**
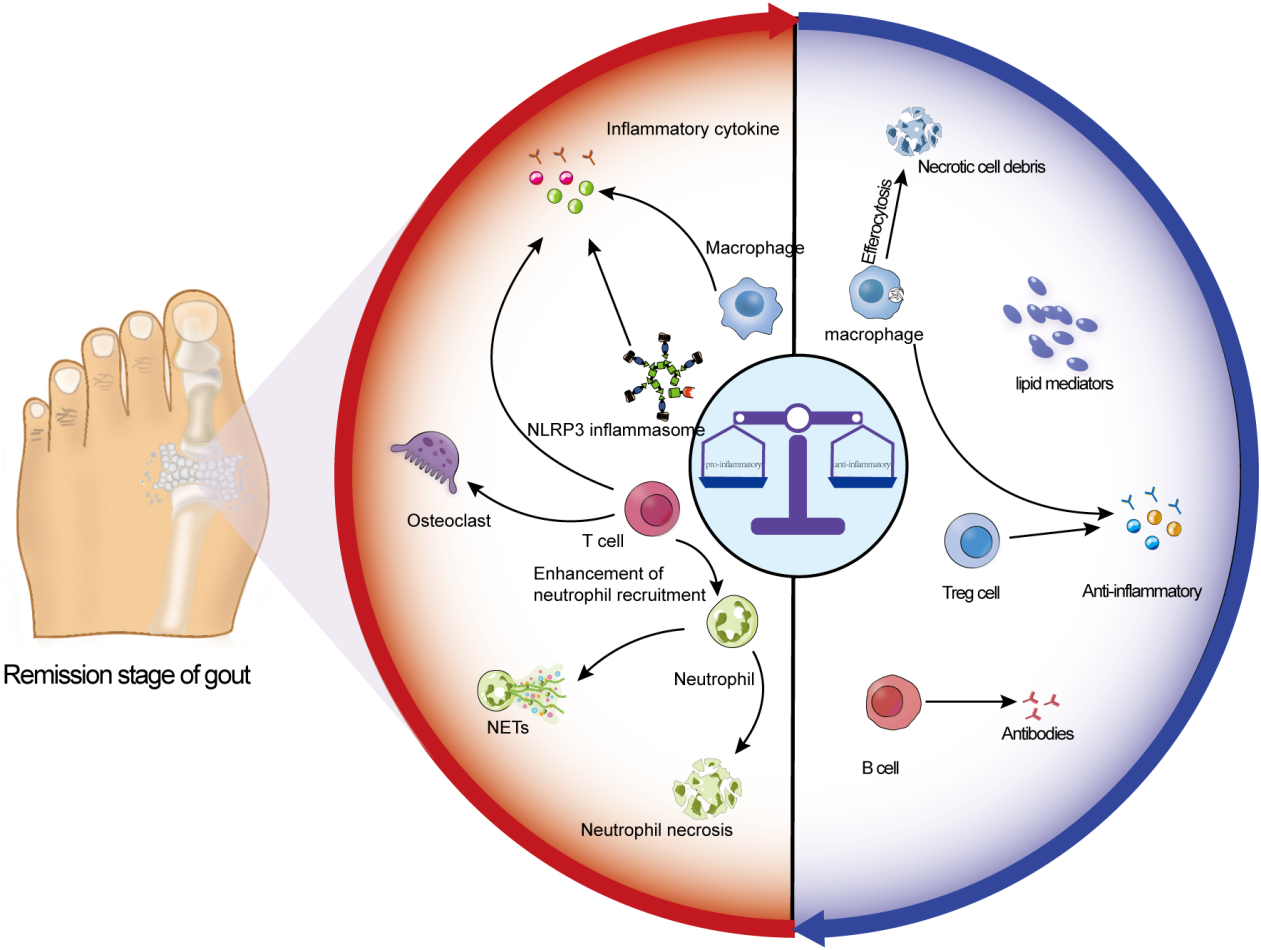
Schematic representation of mechanisms maintaining immune homeostasis during the remission phase of gout: During gout remission, the host does not fully return to a baseline state. Instead, a dynamic equilibrium is maintained through the interplay between low-level local or systemic pro-inflammatory cells and mediators and a range of anti-inflammatory cells and factors. This state constitutes a chronic, low-grade inflammatory environment, commonly referred to as “trained immunity.” In the figure, the red region on the left illustrates pro-inflammatory pathways, whereas the blue region on the right highlights anti-inflammatory regulatory mechanisms. The balance between these opposing forces, shaped by trained immunity, converges toward immune homeostasis and ultimately determines the recurrent nature of clinical manifestations.

In summary, chronic stimulation by MSU crystals may induce persistent functional and epigenetic reprogramming of innate immune cells, resulting in the establishment of trained immunity. This concept provides a novel and coherent theoretical framework for understanding sustained subclinical inflammation during the remission phase of gout and the high propensity for recurrent acute flares (27, 29). However, it must be emphasized that, although this hypothesis is mechanistically plausible and supported by analogous evidence from other chronic inflammatory diseases (39, 40), direct functional evidence in human gout remains in an emerging stage. Future longitudinal studies are required to determine whether immune cells from patients with gout harbor canonical epigenetic signatures and metabolic features of trained immunity, and to clarify their direct causal relationship with disease relapse. Distinguishing robust theoretical hypotheses from human phenomena that remain to be directly validated will be essential for guiding future translational research (**Figure 3**).

## 4 Temporal differences in immune responses in gout: time-dependent changes in immune cells and the IL-1β signaling pathway

### 4.1 Flare phase (24 h)

**Figure 4:**
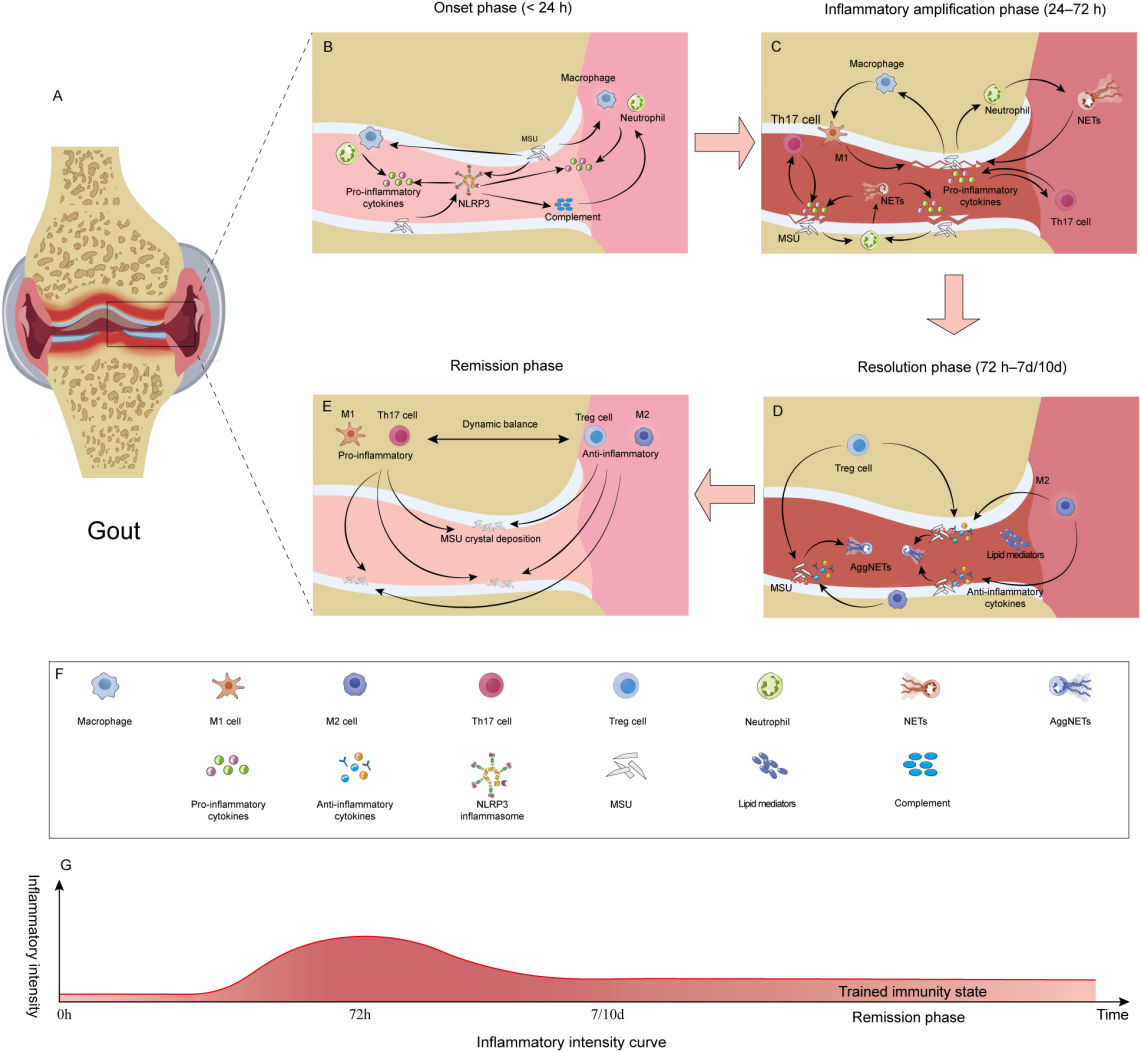
Temporal evolution of immunoinflammatory responses in gouty arthritis: **(A)** Schematic illustration of joint involvement in gouty arthritis. **(B)** Flare phase (<24 h): MSU crystal deposition activates the NLRP3 inflammasome, triggering the initial recruitment of neutrophils and macrophages and activation of the complement system. **(C)** Inflammation amplification phase (24 -72 h): M1-polarized macrophages, Th17 cells, and neutrophils act in concert to produce large amounts of pro-inflammatory mediators, culminating in a peak inflammatory response. **(D)** Resolution phase (>72 h to 7 -10 days): Tregs, M2-polarized macrophages, aggregated aggNETs, and lipid mediators promote active inflammation resolution. **(E)** remission phase: A dynamic equilibrium is established within the joint; despite residual MSU crystals, homeostasis is maintained through a balance between pro- and anti-inflammatory factors. **(F)** Legend detailing the key immune cell subsets, signaling molecules, and microenvironmental components depicted in the figure. **(G)** Dynamic curve of inflammatory intensity across the entire disease course of gout, highlighting the trained immunity state during remission.

In the early stage of an acute gout flare, MSU crystals are initially recognized by synovial monocytes/macrophages. This recognition activates the NF-κB pathway through pattern recognition receptors, including Toll-like receptor 2/4 (TLR2/4) and C-type lectin domain family 12 member A (CLEC12A), thereby providing a “priming” signal for NLRP3 inflammasome assembly (41, 42). Once assembled, the NLRP3 inflammasome induces robust production and release of IL-1β (43, 44). As an immediate innate immune cytokine, IL-1β is critical for initiating host defense responses and further promotes the release of multiple inflammatory mediators (45). At this stage, innate immune cells, including macrophages and neutrophils, are rapidly recruited to the inflamed site, and the sharp increase in IL-1β levels drives severe joint pain and inflammatory responses (46). Studies show that MSU crystals stimulate monocytes/macrophages to secrete IL-1β and TNF-α (47). MSU simultaneously activates the complement system, and the generated C3a and C5a, together with chemokines such as CXCL8/IL-8 and CCL2, cooperatively promote massive neutrophil infiltration (48, 49). This stage is characterized by an IL-1β–centered innate immune response, amplified through the coordinated actions of M1-polarized macrophages and neutrophils. Clinically, antibodies targeting IL-1β, such as canakinumab, are effective in the treatment of acute gouty arthritis (50, 51) (**Figures 4A, B, F, G**).

### 4.2 Inflammation amplification phase (24–72 h)

As the inflammation amplification phase ensues, neutrophil infiltration reaches its peak, and the proportion of polymorphonuclear cells in synovial fluid increases markedly (41). Activated neutrophils undergo degranulation, generate ROS, and release proteolytic enzymes, thereby further damaging local tissues; NETs formed at this stage display pronounced pro-inflammatory properties (52). In parallel, monocytes/macrophages sustain NLRP3 inflammasome activation, continue to produce IL-1β, and exhibit a characteristic M1-polarized phenotype (14, 53, 54).

During this period, T cells also play a prominent role, with Th17 cells becoming particularly active (55). Th17 cells differentiate and mature under the influence of cytokines such as IL-1β, IL-6, and TGF-β (56), and subsequently secrete cytokines including IL-17, IL-21, and IL-22 (57). IL-17 can further promote inflammatory responses, thereby sustaining and amplifying inflammatory signaling (58). Specifically, IL-17 stimulates fibroblasts, endothelial cells, and macrophages to release chemokines such as CXCL8/IL-8 and granulocyte–macrophage colony-stimulating factor (GM-CSF), maintaining neutrophil recruitment and forming a positive feedback loop with the NLRP3–IL-1β axis (59) (**Figures 4C, F, G**).

### 4.3 Resolution phase (>72 h to 7–10 days)

A hallmark of acute gouty arthritis is its tendency to resolve spontaneously within 7–10 days, reflecting the presence of intrinsic inflammatory resolution mechanisms (60). During this phase, large numbers of neutrophils undergo apoptosis and are cleared by macrophages through efferocytosis, driving macrophage polarization from an M1 to an M2 phenotype. M2-polarized macrophages release IL-10, TGF-β, and pro-resolving lipid mediators, thereby suppressing NLRP3 inflammasome activation and promoting tissue repair (61).

At this stage, Tregs play a pivotal role by suppressing inflammatory responses and restoring immune balance through the secretion of immunosuppressive cytokines, including IL-10 and TGF-β. IL-10 is a key anti-inflammatory cytokine that effectively inhibits IL-1β production, and Tregs restrain Th17 cell–mediated inflammation through IL-10–dependent signaling, thereby facilitating the resolution of inflammation (62). At the same time, activated polymorphonuclear neutrophils (PMNs) release NETs, which can entrap MSU crystals and form NET–MSU aggregates. These NET–MSU aggregates are capable of degrading cytokines and chemokines and blocking further neutrophil recruitment and activation, thereby promoting the resolution of gouty inflammation (63, 64). The functional shift of NETs from an early pro-inflammatory role to a later pro-resolving role, together with the coordinated actions of M2-polarized macrophages and Tregs, helps explain the self-limiting nature of acute gouty inflammation (60, 65). In addition, lipid mediator profiling indicates that prostaglandins and leukotrienes predominate in the early phase, whereas resolvins, lipoxins, protectins, and maresins increase during the later phase, giving rise to “lipid mediator class switching, “ which facilitates the termination of inflammation (27, 29) (**Figures 4D, F, G**).

### 4.4 Remission phase

Even after entering clinical remission, the immune system does not become fully “quiescent”. Single-cell transcriptomic studies reveal that, compared with the acute phase, patients in remission exhibit marked changes in the composition of peripheral blood monocyte subsets and the proportion of Tregs, reflecting a state of persistent low-grade activation with features of trained immunity (6). Under conditions of sustained hyperuricemia, monocytes/macrophages are further driven to undergo metabolic and epigenetic reprogramming (66). Based on studies specifically addressing uric acid–induced immune programming, this process may drive the acquisition of a trained immunity phenotype, enabling a heightened inflammatory response upon re-exposure to MSU (27, 29). Accordingly, trained immunity has been proposed as a theoretically attractive potential mechanism underlying the propensity for clinical relapse in gout. Epidemiological studies indicate that gout recurrence is positively correlated with serum urate levels (67). Therefore, to reduce the likelihood of recurrent acute flares, serum urate should be maintained at low levels during the remission phase (**Figures 4E–G**).

## 5 Spatial differences in immune responses in gout: the immune gradient across the joint–bone–circulation axis

The pathological process of gout does not represent a uniform systemic inflammatory response but instead exhibits distinct immune features across different anatomical sites, forming an immune gradient that extends from the local joint to the circulation. This gradient is closely associated with patterns of MSU crystal deposition, the local tissue microenvironment, and systemic immune communication (68).

### 5.1 Intra-articular immune gradient: focal inflammation and structural damage

Intra-articular inflammation is not uniform but instead exhibits a complex spatial distribution, which not only influences disease progression but also provides an additional dimension for mechanistic investigation (69). In gouty arthritis, the synovium represents a key site of inflammatory initiation (70). MSU crystals activate synovial macrophages, neutrophils, and T lymphocytes, accompanied by the release of pro-inflammatory cytokines such as IL-1β, TNF-α, and IL-6 (10). These factors are associated with synovitis, synovial hyperplasia, and angiogenesis in inflammatory joint diseases (71, 72). Cartilage, as a critical structural component of the joint, is also commonly affected by damage in gout (73, 74). Inflammatory mediators and matrix metalloproteinases (MMPs) released by activated synovial cells and chondrocytes drive degradation of the cartilage matrix (73). Such cartilage damage is typically more pronounced in regions with MSU crystal deposition, indicating spatial heterogeneity of local inflammation (74). These differences can be detected using imaging modalities. Ultrasound (US) enables the detection of periarticular MSU deposition, synovitis, and bone erosion (75, 76). Dual-energy computed tomography (DECT) can identify tophus deposition and assess intra-articular bone lesions (77, 78). Magnetic resonance imaging (MRI) is used to evaluate inflammation, bone erosion, and cartilage damage. Together, these imaging techniques reveal the fine spatial distribution of gouty lesions within the joint, reflecting the focal deposition of MSU crystals on the articular cartilage surface and within the synovium (79).

**Figure 5:**
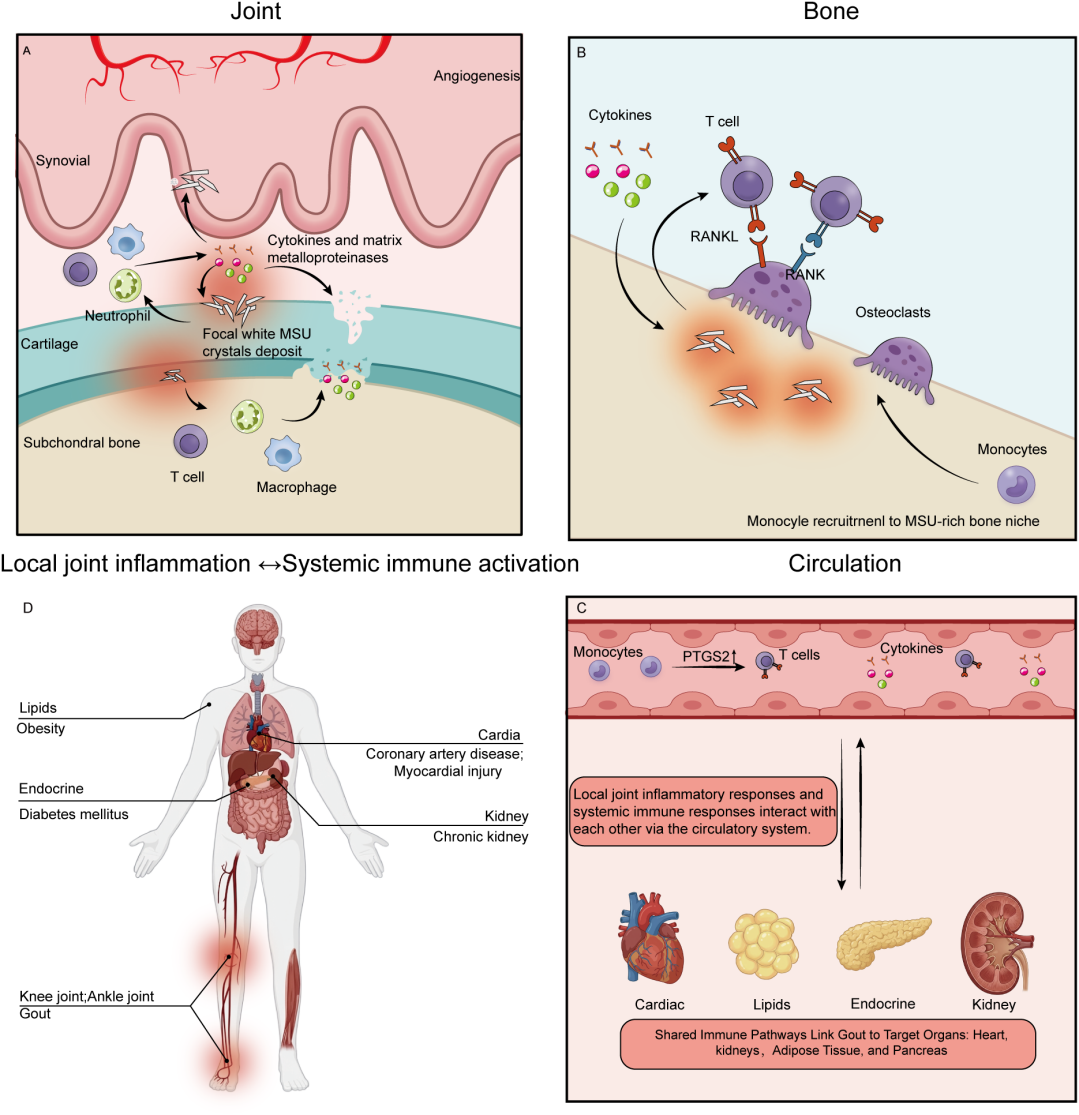
The immune gradient of gout across the joint–bone–circulation axis. **(A)** Joint: This panel illustrates synovial inflammation triggered by deposition of MSU crystals. Recruitment of neutrophils, macrophages, and T cells is evident, accompanied by the release of pro-inflammatory cytokines and MMPs, ultimately leading to angiogenesis and degradation of the cartilage matrix. **(B)** Bone: This panel depicts the impact of the inflammatory microenvironment on bone metabolism. Activation of osteoclasts through the RANKL–RANK signaling pathway promotes monocyte recruitment and results in localized bone erosion. **(C, D)** Circulation: These panels illustrate the evolution of gout from a localized inflammatory process to a systemic condition. Circulating monocytes and cytokines disseminate inflammatory signals through the circulation, establishing pathological links between the joint and distant organs, including the heart, adipose tissue, endocrine system, and kidneys, thereby highlighting the associations between gout and comorbidities such as coronary artery disease, diabetes mellitus, and chronic kidney disease.

These imaging modalities provide macroscopic spatial information, whereas spatial transcriptomics enables the interrogation of tissue-intrinsic spatial heterogeneity at the cellular and molecular levels (80, 81). Although direct spatial transcriptomic data from gouty synovial tissue are still emerging, single-cell studies of peripheral blood and synovial fluid have already uncovered clues to the spatial heterogeneity of immune responses. For example, evidence indicates that even during clinical remission, MSU crystal deposition continues to drive both local and systemic immune responses (24). A single-cell sequencing study of peripheral blood mononuclear cells from patients with gout identified a distinct pro-inflammatory monocyte subset present during remission, whose transcriptomic profile is associated with a systemic inflammatory state (6). More detailed investigations in patients in remission with MSU crystal deposition further demonstrate that crystal deposits directly contribute to shaping the local immune microenvironment, indicating a high degree of spatial specificity in immune cell infiltration and activation within the joint (24). Collectively, these findings suggest that intra-articular inflammation in gout is not diffuse or uniform but instead is organized around sites of crystal deposition, forming a spatial immune gradient mediated by specific immune cell subsets across molecular, cellular, and tissue levels (**Table 1**; **Figure 5A**).

**Table 1:** Summary of key evidence supporting the spatiotemporal immune gradient in gout.

Technique/Method	Samples and key findings	Supported gradient dimension	References
Single-cell RNA sequencing	Peripheral blood PBMCs: Identification of remission-specific pro-inflammatory monocyte subsets and functionally altered Tregs.	Temporal gradient (remission vs acute phase); systemic gradient (circulating immune state)	(6)
Advanced imaging (US/MRI/DECT)	Gouty joints: Visualization of MSU crystal deposition, synovitis, and bone erosion showing focal and heterogeneous distribution.	Spatial gradient (anatomical distribution and heterogeneity)	(75–79)
Theoretical synthesis/review	Systematic integration of immunoinflammatory mechanisms and local–systemic interactions, providing a conceptual framework for the gradient model.	Integrated gradient (spatial–systemic interaction)	(6, 63, 90)

### 5.2 Immune gradient in bone: from periarticular erosion to systemic alterations in bone metabolism

Gout not only affects articular cartilage but also extends to the subchondral bone, ultimately manifesting as bone destruction and erosion (82, 83). From the perspective of osteo–immune interactions, MSU crystals deposited on joint and bone surfaces enhance the activity of the RANKL–osteoclast axis. In addition, certain T cell subsets, upon inflammatory stimulation, secrete RANKL, thereby promoting osteoclast differentiation and activation. Concurrently, pro-inflammatory cytokines such as IL-1β, TNF-α, and IL-17 create a local immunoinflammatory “hotspot, “ which further exacerbates osteoclastogenesis and bone resorption (16). On the one hand, these upregulated inflammatory factors are enriched in pathways related to bone metabolism; in gout patients in remission with persistent MSU crystal deposition, elevated inflammatory mediators may directly participate in processes of bone destruction. On the other hand, monocytes migrating into joints with MSU crystal deposition can differentiate into osteoclasts under inflammatory conditions and the combined influences described above, thereby contributing to bone resorption (84) (**Figure 5B**).

### 5.3 Immune gradient in the circulation: systemic inflammation and a central hub for cellular recruitment

The circulation serves as a critical bridge linking local joint inflammation with systemic immune status by mediating the transport of inflammatory cytokines and molecular signals (85). Even during clinical remission, the systemic immune state of patients with gout does not fully subside. Single-cell transcriptomic analyses provide supportive evidence for this notion: during remission, peripheral blood from patients with gout harbors monocyte subsets with distinct transcriptomic profiles, and both the proportion and functional state of Tregs differ significantly from those observed in healthy controls or in patients during the acute phase (6). In addition, another study further reveals that MSU crystal deposition itself can continuously drive transcriptional reprogramming across multiple immune cell types. Such systemic signals may originate from the local joint microenvironment and propagate throughout the body via circulating immune cells (24). Together, these data support the existence of an immune activation gradient that is transmitted from local crystal deposition sites to the systemic circulation (24, 68).

This systemic state of immune activation constitutes a shared inflammatory basis between gout and multiple comorbid conditions. Given the similarities in immune mechanisms across inflammatory diseases, the persistent activation of peripheral immune cells described above may be closely linked to the high prevalence of cardiometabolic disorders and diabetes observed in patients with gout (86, 87). At the same time, T cell activation plays an important role in the inflammatory responses associated with myocardial ischemia–reperfusion injury, coronary atherosclerotic heart disease, obesity, and diabetes (88, 89). Beyond metabolic disorders, chronic kidney disease is also closely linked to gout (90). On the other hand, chemokines present in the circulation can promote the recruitment of immune cells and inflammatory signals to the joint, thereby triggering local inflammatory responses (91). This indicates that local joint inflammation and systemic immune responses are interconnected through the circulation (**Figures 5C, D**).

### 5.4 Molecular basis of spatially specific immune gradients

The emergence of this spatial immune gradient–associated pattern is rooted in molecular differences in how distinct tissue microenvironments respond to MSU crystals. Within the joint cavity, high local concentrations of MSU directly initiate a canonical innate immune response centered on the NLRP3 inflammasome and the IL-1β axis (17). Concurrently, locally produced chemokines generate concentration gradients that precisely direct the routes of immune cell infiltration (91). In addition, emerging perspectives on neuro–immune interactions warrant attention. Although evidence in gout is still accumulating, sensory nerve endings that detect crystal deposition and pain may release neuropeptides and related mediators, thereby retrogradely modulating local vascular permeability and immune cell function and contributing to the shaping of a three-dimensional inflammatory spatial architecture (92, 93). Overall, immune responses in gout exhibit multi-layered spatial differences and interconnections across the joint, bone, and Circulation. Elucidating these patterns is of critical importance for the development of precision intervention strategies that target specific disease sites, such as the synovium or the bone, as well as for the use of circulating biomarkers in disease staging and prognostic assessment.

## 6 Spatiotemporally stratified precision intervention strategies for gout

Building on the dynamic immunoinflammatory axis that characterizes the entire course of gout, clinical management strategies are shifting from a traditional, nonselective model of “global inflammation suppression” toward a precision intervention paradigm based on temporal stratification and spatial targeting (94). The core of this approach lies in tailoring differential and individualized therapies according to the dominant immunopathological features at distinct disease stages and the anatomical distribution of inflammation, with the aims of enhancing therapeutic efficacy, minimizing systemic adverse effects, and actively restoring immune homeostasis.

### 6.1 Precision interventions based on the temporal evolution of inflammation

#### 6.1.1 Flare phase: targeting the NLRP3–IL-1β axis

MSU crystals activate the NLRP3 inflammasome, driving the maturation and release of IL-1β, which represents the initiating and central event in the acute inflammatory flare of gout (95, 96). IL-1β is not only a potent pro-inflammatory mediator but also a key driver of neutrophil recruitment to the joint (97). Therefore, the therapeutic focus at this stage is to rapidly block IL-1β signaling. The IL-1 receptor antagonist anakinra exerts rapid anti-inflammatory effects by competitively binding to the IL-1 receptor, and has demonstrated clear efficacy in IL-1–driven diseases, such as systemic juvenile idiopathic arthritis (98–100). The fully human anti–IL-1β monoclonal antibody canakinumab directly neutralizes IL-1β. Its long half-life (approximately 28 days) makes it suitable for patients requiring sustained suppression, and it has shown favorable efficacy in conditions such as adult-onset Still’s disease (101, 102). In addition, blocking the interaction between the complement activation fragment C5a and its receptor (C5aR) can inhibit the recruitment of inflammatory cells and may have synergistic potential when combined with IL-1 blockade.

#### 6.1.2 Inflammation amplification phase: suppressing cooperative pro-inflammatory signaling and metabolic reprogramming

As inflammation progresses, cytokines such as IL-6 and IL-17 act synergistically with IL-1β to amplify inflammatory responses and drive tissue damage (103, 104). Accordingly, targeting these cooperative pathways, such as IL-6 or IL-17 signaling, may theoretically confer therapeutic benefit. Case reports and small-scale studies suggest that IL-6 receptor antagonists (such as tocilizumab) or IL-17 inhibitors (such as secukinumab) may be effective in patients with refractory gout or in those exhibiting specific inflammatory profiles, such as elevated IL-6 levels. However, their definitive efficacy and clinical positioning require validation in large-scale clinical trials (105–109). This stage is also accompanied by glycolytic reprogramming of immune cells. Targeting key nodes at the interface of metabolism and inflammation, such as inhibiting rate-limiting enzymes of glycolysis, may attenuate myeloid cell activation and effector functions, thereby weakening the persistence of inflammatory responses at their source.

#### 6.1.3 Resolution phase: actively promoting inflammation resolution and tissue repair

The termination of inflammation is an active and programmed process (36). At this stage, therapeutic goals should shift from “suppression” to the active promotion of resolution. Preclinical studies indicate that administration of analogs of SPMs, such as lipoxins and protectins, or the use of pro-resolving molecules such as annexin A1–derived peptides, may actively facilitate the resolution of inflammation (110–112). These effects are mediated, for example, through enhanced clearance of apoptotic cells and inhibition of neutrophil infiltration, providing a conceptual framework for the development of novel pro-resolving therapies (113, 114). In parallel, enhancing Treg function represents a key strategy. Low-dose IL-2 can selectively expand Tregs, which suppress Th1/Th17 responses through the secretion of IL-10 and TGF-β, thereby accelerating inflammation resolution and tissue repair (50, 115).

#### 6.1.4 Remission phase: modulating trained immunity to prevent recurrence

During clinical remission, innate immune cells may undergo epigenetic reprogramming that establishes a state of trained immunity, maintaining low-grade activation that serves as a potential substrate for disease recurrence and may also increase cardiovascular risk (39, 40). Accordingly, the therapeutic objective during this phase is to modulate aberrant trained immunity and restore immune homeostasis. Based on the concept that trained immunity involves epigenetic reprogramming, emerging evidence suggests that epigenetic modulators, such as histone deacetylase (HDAC) inhibitors, may have potential therapeutic value (116). However, the efficacy of such strategies in gout remains speculative and requires experimental validation.

### 6.2 Precision interventions based on the spatial distribution of inflammation: localized delivery and dynamic monitoring

#### 6.2.1 Local targeted drug delivery systems

Given that the clinical manifestations of gout inflammation are often confined to the joint, the development of local drug delivery systems represents an ideal approach for achieving spatially precise intervention while minimizing systemic exposure. Local injection of nano-carriers or liposomal formulations can enable high-concentration and sustained delivery of agents such as IL-1β inhibitors to the synovial and cartilage compartments, thereby markedly enhancing local efficacy and reducing systemic adverse effects. For example, epigallocatechin gallate has been shown to inhibit NLRP3 inflammasome activation when administered locally and to exhibit potential in preventing acute flares in experimental models (117).

#### 6.2.2 Biomarker-based dynamic monitoring and guidance

Achieving precision intervention depends on real-time assessment of the local inflammatory state. Dynamic monitoring of synovial fluid, with quantitative measurement of key mediators such as IL-1β, IL-6, and NETs, enables objective evaluation of local inflammatory activity and provides direct guidance for the selection and adjustment of local or systemic therapeutic strategies. In addition, the application of metabolomic approaches to analyze synovial fluid metabolic profiles may help identify specific metabolic pathways that drive local inflammation, thereby uncovering novel targets for localized therapy. The integration of local targeted drug delivery with real-time biomarker monitoring establishes a closed-loop precision management framework for assessment and intervention.

## 7 Conclusion

Gout is not a simple crystal deposition disease but rather an immunoinflammatory disorder that evolves in a highly dynamic manner across both temporal dimensions (recurrent cycles of flare, inflammation amplification, resolution, and remission) and spatial dimensions (the local joint, bone, and circulation). The “spatiotemporal immune gradient” framework reviewed in this article integrates diverse lines of evidence ranging from clinical observations to molecular mechanisms, providing a systematic perspective for understanding this complexity. This framework reveals that the pathological process of gout is driven by persistent MSU crystal stimulation and sustained by a dynamic network involving coordinated interactions among immune cells and inflammatory mediators.

However, it must be acknowledged that several key controversies and unresolved questions remain within this framework and require clarification by future studies. First, although IL-1β occupies a central position, the independent contributions of other cytokines, such as TNF-α, IL-6, and IL-17, as well as their interactions with the IL-1 axis, have not been fully delineated. This complexity may underlie the interindividual variability observed in the clinical efficacy of IL-1–targeted therapies. Second, the identity of the “initiating” cells responsible for acute flares—whether tissue-resident macrophages or rapidly recruited monocytes—remains a subject of debate, with direct implications for the precise selection of preventive therapeutic targets. Finally, concepts such as trained immunity and spatial immune heterogeneity, although supported by emerging evidence, still require validation through longitudinal studies and spatial omics data obtained directly from human subjects. More fundamentally, many of the finely delineated immune mechanisms discussed herein, including the regulation of NLRP3 activation and the specialized functions of distinct immune cell subsets, were initially characterized in animal models. Their exact relevance, relative weight, and modes of manifestation in human gout pathophysiology remain to be definitively confirmed and translated using patient-derived samples (**Table 2**).

**Table 2:** Evidence sources and human validation status of key immunological mechanisms in gout.

Key mechanistic step	Primary evidence source (experimental models)	Validation status in human gout	References
1. MSU crystal–induced activation of the NLRP3 inflammasome	Murine macrophage systems; peritoneal inflammation models	Partially validated: Activation of NLRP3 and IL-1β production have been demonstrated in patient-derived monocytes/macrophages.	(4, 13, 18)
2. Central pro-inflammatory role of IL-1β	Murine peritonitis models; IL-1R/IL-1β knockout mice	Strongly validated: Elevated IL-1β levels in patients; clinical efficacy of IL-1–targeted therapies.	(19, 50, 51)
3. Neutrophil NETosis	Murine air pouch models; *in vitro* experiments	Preliminarily validated: NETs and aggregated NET structures detected in synovial fluid from patients.	(15, 52, 63, 64)
4. Th17/IL-17 pathway involvement	MSU-induced immunization mouse models	Supportive evidence: Increased Th17 cell proportions and elevated IL-17 levels in patients.	(16, 55, 57)
5. Development of “trained immunity”	Trained macrophage models in mice	Preliminary/indirect evidence: Transcriptomic and epigenetic alterations observed in patient monocytes.	(6, 24, 28, 29)
6. Local spatial inflammatory gradients	Limited animal model evidence	Primarily supported by human studies: Imaging and single-cell sequencing reveal focal and heterogeneous distribution.	(6, 24, 73, 78)

Looking ahead, continued dissection of the spatiotemporal principles governing immune responses in gout is expected to drive a paradigm shift in clinical management from “global inflammation suppression” toward precision intervention. Temporally informed strategies require the identification and targeting of dominant mechanisms at distinct disease stages, such as the inflammatory storm during flares and immune memory–like states during remission. Spatially informed interventions, in turn, call for the development of therapies that selectively target local joint inflammation or protect the bone, while simultaneously monitoring and managing the comorbidity risks associated with systemic immune activation. Only by confronting existing controversies and deepening our spatiotemporal understanding can we ultimately achieve a transition in gout care from symptomatic control to true disease modification.
